# Evaluation of the Effects of Chitin and Chitosan on Pseudo‐Allergic Reaction by Inhibiting MRGPRX2 Activation

**DOI:** 10.1002/fsn3.70877

**Published:** 2025-09-01

**Authors:** Tao Jia, Ruiqi Li, Chenkun Liu, Yifan Xia, Mengyao Yi, Xiangjin Song, Tong Zhou, Delu Che, Ning Kong

**Affiliations:** ^1^ Department of Dermatology The Second Affiliated Hospital of Xi'an Jiaotong University Xi'an China; ^2^ Bone and Joint Center The Second Affiliated Hospital of Xi'an Jiaotong University Xi'an China

**Keywords:** cytokines, degranulation, inflammation, mast cells

## Abstract

Chitin and chitosan, characterized by their extensive applications, abundant availability, and low cost, have been demonstrated to modulate immune responses. Mast cells (MCs) are important innate immune cells, and few studies on the regulation of MCs by chitin and chitosan were conducted. The key receptor Mas‐related G protein–coupled receptor X2 (MRGPRX2), highly expressed in MCs, is involved in drug pseudo‐allergic responses and several chronic diseases by mediating MC activation. However, the inhibitory effects of chitin and chitosan on MRGPRX2 activation of MCs have not been reported. To verify whether chitin and chitosan inhibit MRGPRX2‐mediated MCs activation and determine which of these compounds shows the best inhibitory effect, in vitro MC degranulation reaction and in vivo substance P (SP)‐induced local passive anaphylaxis models were used to evaluate the inhibitory effect of chitin and chitosan on MRGPRX2 activation of MCs. We showed that both chitin and chitosan inhibited MC degranulation mediated by MRGPRX2 and reduced β‐hexosaminidase, histamine, TNF‐α, MCP‐1, and IL‐8 release in vitro. Chitin and chitosan inhibit local pseudo‐allergic reactions and reduce microvascular dilation by inhibiting MRGPRX2‐mediated MC activation. Both chitin and chitosan inhibited MRGPRX2‐mediated MC degranulation. However, chitosan showed a stronger inhibitory effect. Chitosan has the potential to be incorporated into functional foods as an auxiliary in the treatment of MRGPRX2‐mediated chronic diseases.

## Graphical abstract

Our study suggested that chitin and chitosan could inhibit MRGPRX2/MrgprB2‐mediated mast cells degranulation in vitro and in vivo. Chitosan showed stronger inhibition effect on MRGPRX2 activation than chitin. RNA‐seq analysis indicated that it may affect the activity of structural molecules, chromatin structural components and protein heterodimerization activity.

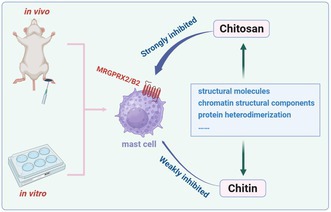

## 1 Introduction

Chitin and chitosan, which derive from the shells and exoskeletons of a variety of marine and nonmarine species, are polymers that are abundant in nature (Satitsri and Muanprasat 2020). Chitin is a long‐chain polymer of N‐acetylglucosamine that is found in the skeletal structures of a wide range of unicellular and multicellular organisms, including diatoms, protists, fungi, sponges, corals, mollusks, worms, and arthropods (Tsurkan et al. 2021). Chitin is one of the most fundamental structural biopolymers in animals and has a long history (Bengtson et al. 2017; Loron et al. 2019). In nature, chitin biomaterials are formed by nanochitin‐protein, chitin‐pigment, and chitin‐mineral complexes. Chitosan is a deacetylated chitin derivative mainly composed of D‐glucosamine. Chitosan derivatives can be obtained by modifying their functional groups to improve the physicochemical properties of molecule while retaining its characteristics and expanding the scope of its applications (Razmi et al. 2019; Ren et al. 2019).

Biodegradability, biocompatibility, non‐toxicity, and non‐immunogenicity are among the advantages of chitin and chitosan (Satitsri and Muanprasat 2020; Sivanesan et al. 2021). Owing to this biocompatibility, the polycationic polymer of chitosan can form ionic and hydrogen bonds with many drug molecules and, therefore, it is used in drug delivery systems (Parhi 2020; Zheng et al. 2021). The development of chitosan nanoparticles has greatly increased their use in the local delivery of drugs (Fonseca‐Santos and Chorilli 2017; Mohammed et al. 2017). Chitin also has anticancer effects; in addition to the delivery of anticancer drugs, it can induce anti‐tumor proliferation by reducing cell viability (G. Wang et al. 2021). Chitosan and its derivatives are also used in wound dressings due to their ability to promote rapid epithelial cell and collagen production in fibroblasts (Huang et al. 2014; Jafari et al. 2020).

MRGPRX2 is highly expressed on mast cells (MCs), which consist of 330 amino acids (Alkanfari et al. 2018) and can be stimulated by various endogenous or exogenous substances with cationic properties to activate MCs, such as quinolone, substance P (SP), LL‐37, and so on, which lead to the release of various inflammatory mediators (Kühn et al. 2021). Pseudo‐allergic reactions are those reactions that induce allergy‐like symptoms, but without any potential antigen‐specific sensitization or elevated IgE levels. The symptoms include local erythema and itching that occur rapidly after exposure to the sensitizing agent, especially after subcutaneous injections, and may subside after drug withdrawal. Pseudo‐allergic sensitizers, including peptides or small‐molecule drugs, can act directly on MRGPRX2 and activate MCs to produce a degranulation response (McNeil et al. 2015; Subramanian et al. 2016).

Chitin and chitosan play a role in the pathogenesis of inflammatory and respiratory diseases such as asthma. However, their ability to act on MCs and whether they can act on MRGPRX2 is unknown. We investigated the effects of chitin and chitosan on MCs and their mechanisms of action by introducing a human MC line, performing toe swelling experiments, and exploring the roles of these molecules in anaphylactoid reactions to provide a basis for the use of chitin and chitosan in the treatment of inflammatory diseases.

## 2 Materials and Methods

### 2.1 Reagents

Positive control substance P (SP) was purchased from MCE (Shanghai, China, CAS No: 33507‐63‐0). MrgprX2 antagonist‐1 was purchased from MCE (Shanghai, China, CAS No: 2642162‐06‐7). Chitin from shrimp shells (CAS No: 1398‐61‐4) and chitosan from shrimp shells (CAS No: 9012‐76‐4, deacetylation: 83%, molecular weight: 190‐376 kDa) were from Sigma‐Aldrich (Shanghai, China). Sodium carboxymethyl cellulose (CMC‐Na, Viscosity: 800–1200 mPa⋅s, CAS No.: 9004‐32‐4) was purchased from MCE (Shanghai, China).

Add 0.5 g of CMC‐Na powder to 80 mL of StemPro‐34 medium, Tyrode's solution, or saline and dissolve it using ultrasonic heating. After dissolution, transfer the solution to a centrifuge tube. Then, continue to add StemPro‐34 medium, Tyrode's solution, or saline until the total volume reaches 100 mL. The CMC‐Na solution was prepared before use. Chitin and chitosan were added to the CMC‐Na solution and treated by ultrasonic heating. In vitro, chitin and chitosan were prepared with the StemPro‐34 medium or Tyrode's solution with 0.5% CMC‐Na. In vivo, chitin and chitosan were prepared with the saline with 0.5% CMC‐Na.

### 2.2 Cell Lines

The Laboratory Allergic Disease 2, LAD2 cells were kindly provided by A. Kirshenbaum and D. Metcalfe (NIH, USA). The cell culture medium was StemPro‐34 medium, which contained StemPro nutrient supplement (10 mg/L), human SCF (100 ng/mL), and L‐glutamine (2 mmol/L) and the cells were cultured in a cell incubator at 37°C, 5% CO_2_. LAD2 cells that have been passaged within 8 generations were used for the experiment.

### 2.3 Cytotoxicity Assays

1 × 10^5^ LAD2 cells were seeded into a 96‐well plate and treated with 100 μL chitin and chitosan (6.25, 12.5 and 25 μg/mL) prepared with StemPro‐34 medium and 0.5% CMC‐Na. The LAD2 cells treated with StemPro‐34 medium and 0.5% CMC‐Na were set as a negative control, and the experiment was repeated 3 times. The cells were cultured in a cell incubator at 37°C, 5% CO_2_ for 24 h. Then, 10 μL of Cell Counting Kit solution was added to each well and incubated for 4 h. Abbkine‐Cell Counting Kit solution (10 μL, California, USA) was added, incubated for 4 h, and detected at an absorbance of 450 nm.

### 2.4 Histamine and β‐Hexosaminidase Release Assay

1 × 10^5^ LAD2 cells were incubated with 100 μL chitin and chitosan (6.25, 12.5 and 25 μg/mL) prepared by Tyrode's solution with 0.5% CMC‐Na for 30 min in a cell incubator at 37°C, 5% CO_2_. Only Tyrode's solution buffer with 0.5% CMC‐Na without chitin and chitosan was set as a vehicle control group, and SP prepared by Tyrode's solution with 0.5% CMC‐Na was set as the positive control group. The normalization was handled by the volume of supernatant.

To inhibit SP‐induced histamine and β‐hexosaminidase release, LAD2 cells were incubated with 100 μL chitin and chitosan (6.25, 12.5 and 25 μg/mL) by prepared Tyrode's solution buffer with 0.5% CMC‐Na for 30 min in a cell incubator at 37°C, 5% CO_2_ and then centrifuged to remove the supernatants. The 16 μg/mL SP (100 μL) prepared in Tyrode's solution buffer was added in the well for 30 min in a cell incubator at 37°C, 5% CO_2_ and then centrifuged to collect the supernatants. The release of histamine and β‐hexosaminidase was analyzed by ELISA kits (mlbio Biotechnology, Shanghai, China). 1 μM MrgprX2 antagonist‐1 was used as a positive control, and Tyrode's solution buffer with 0.5% CMC‐Na was set as a vehicle control group. The normalization was handled by the volume of supernatant.

For the IC_50_ value analysis, the β‐hexosaminidase release was analyzed after chitin and chitosan treatment by ELISA kit. The experiment was repeated three times. The data was conducted using GraphPad Prism 10.0 software by nonlinear regression to obtain the fitting curve and IC_50_ value.

### 2.5 Chemokine Release Assay In Vitro

The LAD2 cells were incubated with 100 μL chitin and chitosan (6.25, 12.5 and 25 μg/mL) containing 16 μg/mL SP prepared in Tyrode's solution with 0.5% CMC‐Na. 1 μM MrgprX2 antagonist‐1 was used as a positive control, and Tyrode's solution buffer with 0.5% CMC‐Na was set as a vehicle control group. The cells incubated for 12 h in a cell incubator at 37°C, 5% CO_2_. Collect the supernatant of the cell culture and detect the concentration of cytokines by ELISA. The normalization was handled by the volume of supernatant. Human tumor necrosis factor (TNF)‐α, monocyte chemoattractant protein (MCP)‐1 and interleukin (IL)‐8 ELISA Kits were purchased from Sino Bio CO. LTD. (Beijing, China).

### 2.6 Mice

The 8 weeks male C57BL/6 mice were from Xi'an Jiaotong University Experimental Animal Center. Mice were divided randomly into the experimental groups (6 mice per group, *n* = 6). The experimental protocols for the mouse model were approved by the Animal Ethics Committee at Xi'an Jiaotong University (Permit Number: XJTUAE2023‐1175).

### 2.7 Hind Paw Swelling

The C57BL/6 mice were treated with chitin and chitosan (25, 50 and 100 mg/kg) prepared by saline with 0.5% CMC‐Na every 12 h for once by intragastric administration. The mice treated with saline containing 0.5% CMC‐Na were set as a negative control group. After 24 h, the mice were anesthetized using an intraperitoneal injection of 60 mg of barbiturate (Pelltobarbitalum Natricum), followed by a tail vein injection of 0.2 mL of 0.4% Evan's blue (saline preparation). Paw thickness before treatment was measured using a vernier caliper. The left paws were injected with 16 μg/mL SP to activate Mrgprb2, and an equal volume of saline was injected into the right paws as a blank control. The thickness of each paw was measured after 15 min. Then, the tissues were collected. After being dried at 50°C for 12 h, the tissues were weighed. Tissues were extracted in 500 μL acetone‐saline (7:3). The tissues were cut into pieces and centrifuged to extract the Evans Blue at 620 nm.

### 2.8 Skin HandE (Hematoxylin–Eosin) and Avidin Staining

The C57BL/6 mice were treated with chitin and chitosan (25, 50 and 100 mg/kg) prepared by saline with 0.5% CMC‐Na every 12 h once by intragastric administration. The mice treated with saline and 0.5% CMC‐Na were set as a negative control group. Twenty‐four hour later, the mice were anesthetized with Pelltobarbitalum Natricum (60 mg/kg). The left paws were injected with 16 μg/mL SP to activate Mrgprb2. An equal volume of saline was injected into the right paws as a blank control. Then the skin of the paw was collected. The skin was fixed with 4% formaldehyde for 48 h for HandE staining. 1/500 avidin‐FITC antibody was used for marking MCs.

### 2.9 Medium Analysis of Mouse Serum

The male C57BL/6 mice (8 weeks) were treated with chitin and chitosan (25, 50 and 100 mg/kg) prepared by saline with 0.5% CMC‐Na every 12 h for once by intragastric administration. The mice treated by saline with 0.5% CMC‐Na were set as a negative control group. SP injected intravenously and not treated by chitin and chitosan was set as the control group (*n* = 6). Twenty‐four hour later, 30 μg/mL SP prepared by saline was injected intravenously. The blood samples were collected and centrifuged for collecting serum. The medium in mice serum was analyzed by ELISA. The normalization was handled by the volume of supernatant. The mouse histamine, TNF‐α, MCP‐1, and C‐X‐C motif ligand (CXCL) 2 ELISA Kits were purchased from (Meilian Biotechnology, Shanghai, China) and executed according to provided instructions.

### 2.10 RNA‐Seq Analysis

LAD2 cells were treated by 25 μg/mL chitin and chitosan with 16 μg/mL SP for 1 h, and only 16 μg/mL SP treated LAD2 cells were set as the control group. Total RNA of different groups of LAD2 cells (*n* = 3) were collected by TRIzol method. Transcriptomics was analyzed by RNA‐seq (Berry Genomics Corporation, Beijing, China). The statistical power of this experimental design, calculated in NCBI BioProject (ID: PRJNA1179930. https://www.ncbi.nlm.nih.gov/bioproject/?term=PRJNA1179930) was DESeq2. The sequencing data analysis, gene ontology (GO) analysis and Kyoto Encyclopedia of Genes and Genomes (KEGG) analysis, were performed using R. DEG thresholds in RNA‐seq was *|*Log FC*| >* 1.

### 2.11 Statistical Analysis

Data are expressed as mean ± SD. An independent samples analysis of variance was used to determine statistical significance in comparisons of the data using the SPSS software. For the paired comparison samples, the treated groups were compared with the negative control group. Paired *t*‐test was used, and differences were considered significant at *p < 0.05, **p < 0.01, and ***p < 0.005. For the multiple dose samples, one‐way ANOVA was used, and the treated groups were compared respectively with the negative control group.

## 3 Results

### 3.1 Chitin and Chitosan Inhibited SP‐Induced MC Activation In Vitro

**FIGURE 1:**
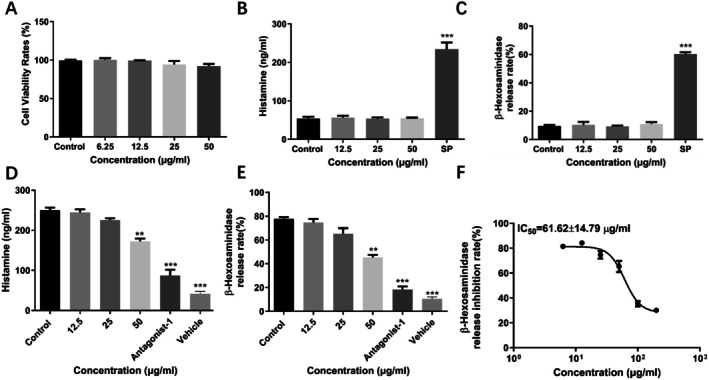
Chitin inhibited MRGPRX2‐mediated LAD2 cells degranulation reaction. (A) Chitin showed little cytotoxicity on LAD2 cells analyzed by Abbkine‐Cell Counting Kit assays. (B and C) Chitin did not induce histamine and β‐hexosaminidase analyzed by ELISA. (D and E) Chitin inhibited MRGPRX2‐mediated LAD2 cells release histamine and β‐hexosaminidase in a dose‐dependent manner analyzed by ELISA. (F) The IC_50_ value of chitin inhibited MRGPRX2‐mediated LAD2 cells release β‐hexosaminidase analyzed by ELISA. (Data expressed as mean ± SD. One‐way ANOVA was used, and the treated groups compared respectively with the negative control group).

**FIGURE 2:**
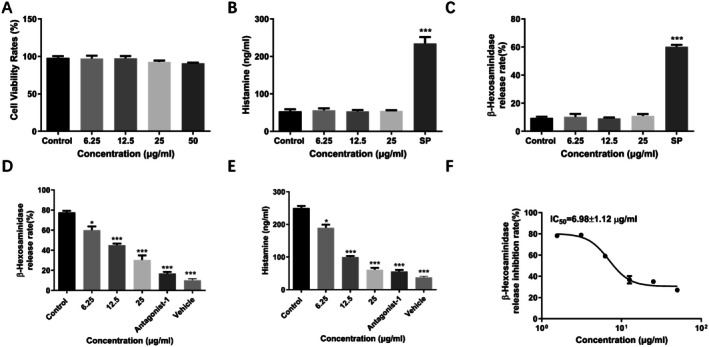
Chitosan inhibited MRGPRX2‐mediated LAD2 cells degranulation reaction. (A) Chitosan showed little cytotoxicity on LAD2 cells analyzed by Abbkine‐Cell Counting Kit assays. (B and C) Chitosan did not induce histamine and β‐hexosaminidase analyzed by ELISA. (D and E) Chitosan inhibited MRGPRX2‐mediated LAD2 cells release histamine and β‐hexosaminidase in a dose‐dependent manner analyzed by ELISA. (F) The IC_50_ value of Chitosan inhibited MRGPRX2‐mediated LAD2 cells release β‐hexosaminidase analyzed by ELISA. (Data expressed as mean ± SD. One‐way ANOVA was used, and the treated groups compared respectively with the negative control group).

The cytotoxic effect on LAD2 cells was first evaluated to evaluate the inhibition effect of chitin and chitosan on MC activation and determine the dosage. The results showed that chitin and chitosan showed over 95% cell viability on LAD2 cells at a concentration of 25 μg/mL (Figures 1A and 2A). In addition, chitin and chitosan did not induce LAD2 cell degranulation reaction and induced little histamine (Figures 1B and 2B) and β‐hexosaminidase release (Figures 1C and 2C).

**FIGURE 3:**
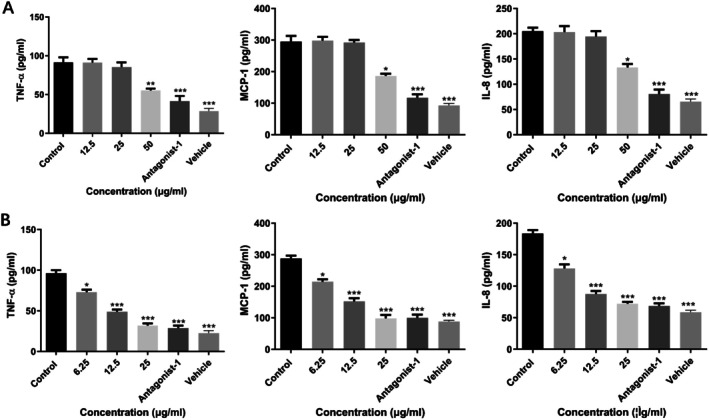
Chitin and chitosan inhibited MRGPRX2‐mediated LAD2 cells release of cytokines. (A) Chitin inhibited MRGPRX2‐mediated LAD2 cells release of TNF‐α, MCP‐1, and IL‐8 analyzed by ELISA. (B) Chitosan inhibited MRGPRX2‐mediated LAD2 cells release of TNF‐α, MCP‐1, and IL‐8 analyzed by ELISA. (Data expressed as mean ± SD. One‐way ANOVA was used, and the treated groups compared respectively with the negative control group).

The inhibitory effect of chitin and chitosan on MRGPRX2‐mediated MC degranulation caused by SP was investigated. We analyzed the cytotoxicity of chitin and chitosan at first, and the dose was chosen under the concentrations which showed little cytotoxicity on LAD2 cells. Then we used mast cell degranulation reaction and set a series concentration of chitin and chitosan, containing 1.5625, 3.125, 6.25, 12.5, 25, 50 μg/mL in vitro. The β‐hexosaminidase release was analyzed to show the inhibitory effect of different concentrations of chitin and chitosan. The data showed that chitin showed little effect under the concentration of 25 μg/mL, and chitosan showed little effect under the concentration of 6.25 μg/mL. We chose the data of final concentration to demonstrate the dose‐dependent effect of chitin and chitosan. It was suggested that chitin and chitosan prevented SP‐induced MC degranulation reaction in vitro in a dose‐dependent manner. The inhibitory effect of chitosan is more obvious. Treated with 50 μg/mL chitin and 25 μg/mL chitosan respectively inhibited the release of histamine from 250.1 ± 6.208 to 172.0 ± 7.259 ng/mL and to 61.10 ± 5.408 ng/mL in vitro (Figures 1D and 2D). Treated with 50 μg/mL chitin and 25 μg/mL chitosan also inhibited the release of β‐hexosaminidase from 77.68 ± 1.500% to 45.35 ± 2.205 and 30.34 ± 4.483% in vitro (Figures 1E and 2E). Moreover, chitin inhibitedβ‐hexosaminidase release with an IC_50_ value of 61.62 ± 14.79 μg/mL (Figure 1F). While chitosan showed a stronger inhibition effect on β‐hexosaminidase release with an IC_50_ value of 6.98 ± 1.12 μg/mL (Figure 2F). In addition, both chitin and chitosan could reduce TNF‐α, MCP‐1, and IL‐8 release in LAD2 cells, and chitosan showed a stronger inhibition effect on cytokines release. Treated with 50 μg/mL chitin respectively reduced TNF‐α, MCP‐1, and IL‐8 release from 91.36 ± 6.558 to 55.35 ± 2.205 pg/mL, 295.1 ± 17.64 to 186.5 ± 7.063 pg/mL, and 205.3 ± 6.549 to 133.3 ± 6.497 pg/mL (Figure 3A). Treated with 25 μg/mL chitosan respectively reduced TNF‐α, MCP‐1, and IL‐8 release from 96.22 ± 3.658 to 31.96 ± 2.515 pg/mL, 288.5 ± 8.303 to 98.10 ± 10.84 pg/mL, and 183.6 ± 5.298 to 72.21 ± 3.073 pg/mL (Figure 3B).

### 3.2 Chitin and Chitosan Inhibit Mrgprb2‐Mediated Anaphylactic Reactions In Vivo

**FIGURE 4:**
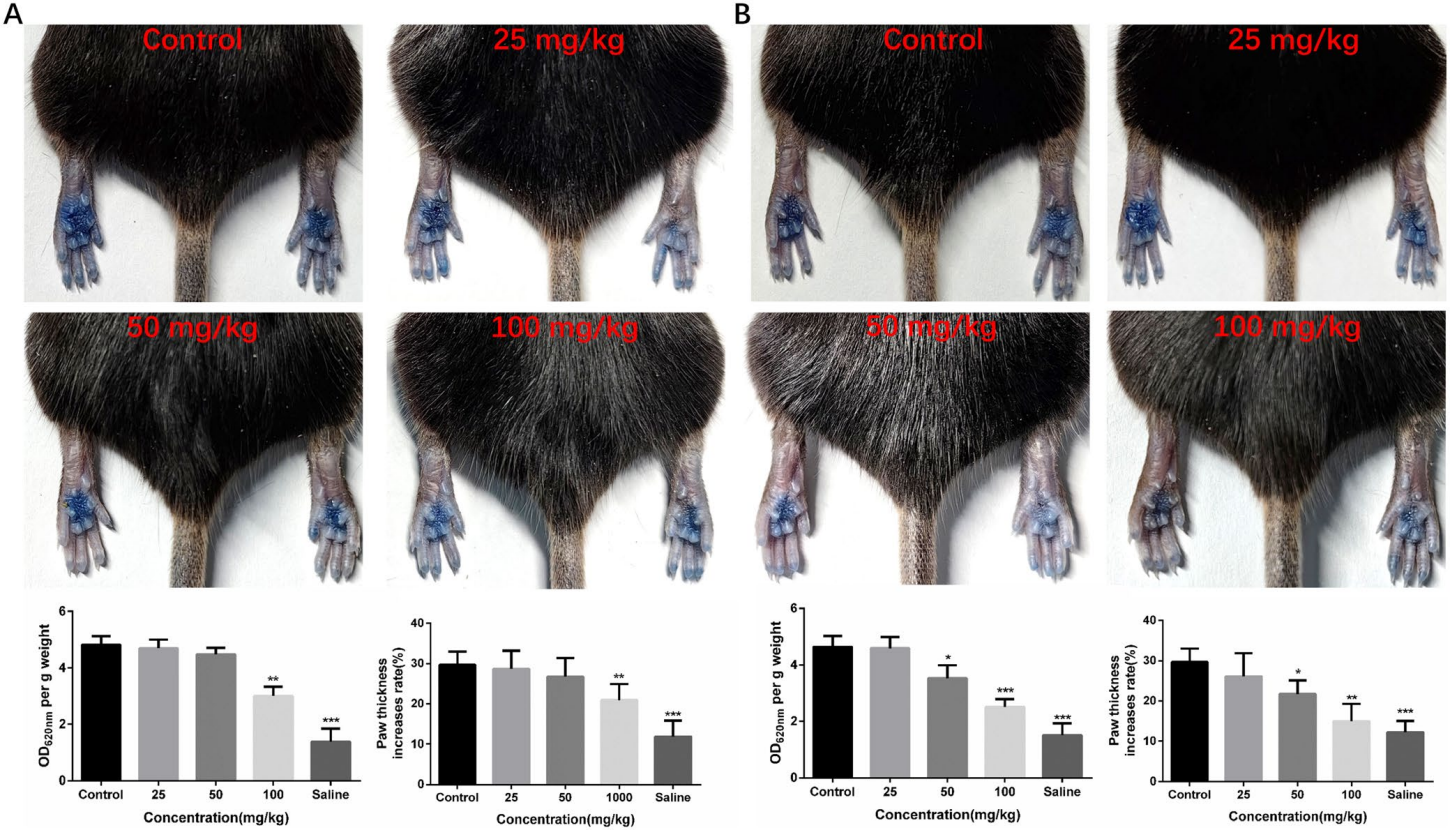
Chitin and chitosan inhibited Mrgprb2‐mediated pseudo‐allergic reaction in mice. (A) Chitin inhibited Mrgprb2‐mediated toe swelling rate and Evans blue exudation with a dose‐dependent manner. (B) Chitosan inhibited Mrgprb2‐mediated toe swelling rate and Evans blue exudation with a dose‐dependent manner, (*n* = 6, Data expressed as mean ± SD. One‐way ANOVA was used, and the treated groups compared respectively with the negative control group).

**FIGURE 5:**
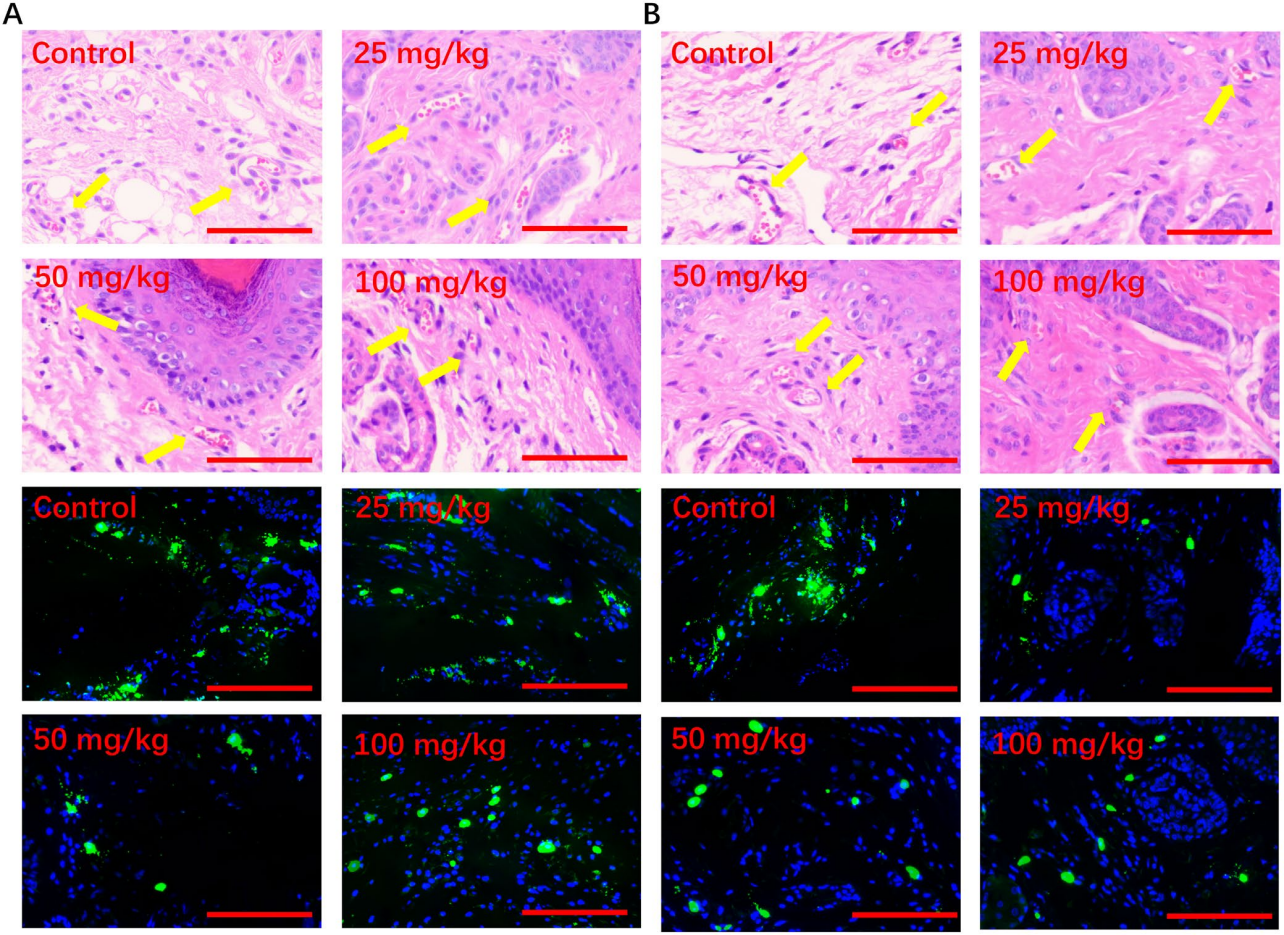
Chitin and chitosan inhibited Mrgprb2‐mediated microangiectasia and MC activation in mice. (A) Chitin inhibited microangiectasia analyzed by HandE staining and inhibited MC activation analyzed by avidin staining. (B) Chitosan inhibited microangiectasia analyzed by HandE staining and inhibited MC activation analyzed by avidin staining. (*n* = 6).

SP‐induced local passive anaphylaxis mouse model via Mrgprb2 was used to verify the inhibitory effect of chitin and chitosan on MC degranulation in vivo. We used a local passive allergic reaction model and set a series concentration of chitin and chitosan, containing 6.25, 12.5, 25, 50, and 100 mg/kg in vivo. The Evans blue exudation was analyzed to determine the dosage concentration of chitin and chitosan. At the doses of 25, 50, and 100 mg/kg, chitin and chitosan showed an inhibitory effect on Mrgprb2‐mediated local passive allergic reaction in a dose‐dependent manner. Treatment with chitin and chitosan could significantly decrease toe swelling rate and Evans blue exudation in mice. Chitosan showed the strongest inhibition effect on the local passive anaphylaxis mouse model at the same concentration as chitin (Figure 4A,B). Pathological examination confirmed that chitin and chitosan reduced SP‐induced microangiectasia by inhibiting skin MC degranulation, and the MC response ratio was significantly decreased after treatment with chitosan (Figure 5A,B). Chitosan showed a stronger inhibition effect on MC degranulation than chitin. MC degranulation was significantly inhibited at the concentration of 50 mg/kg chitosan, and the MC morphology was more regular than control group analyzed by avidin‐FITC, while chitin showed a significantly inhibited at the concentration of 100 mg/kg.

**FIGURE 6:**
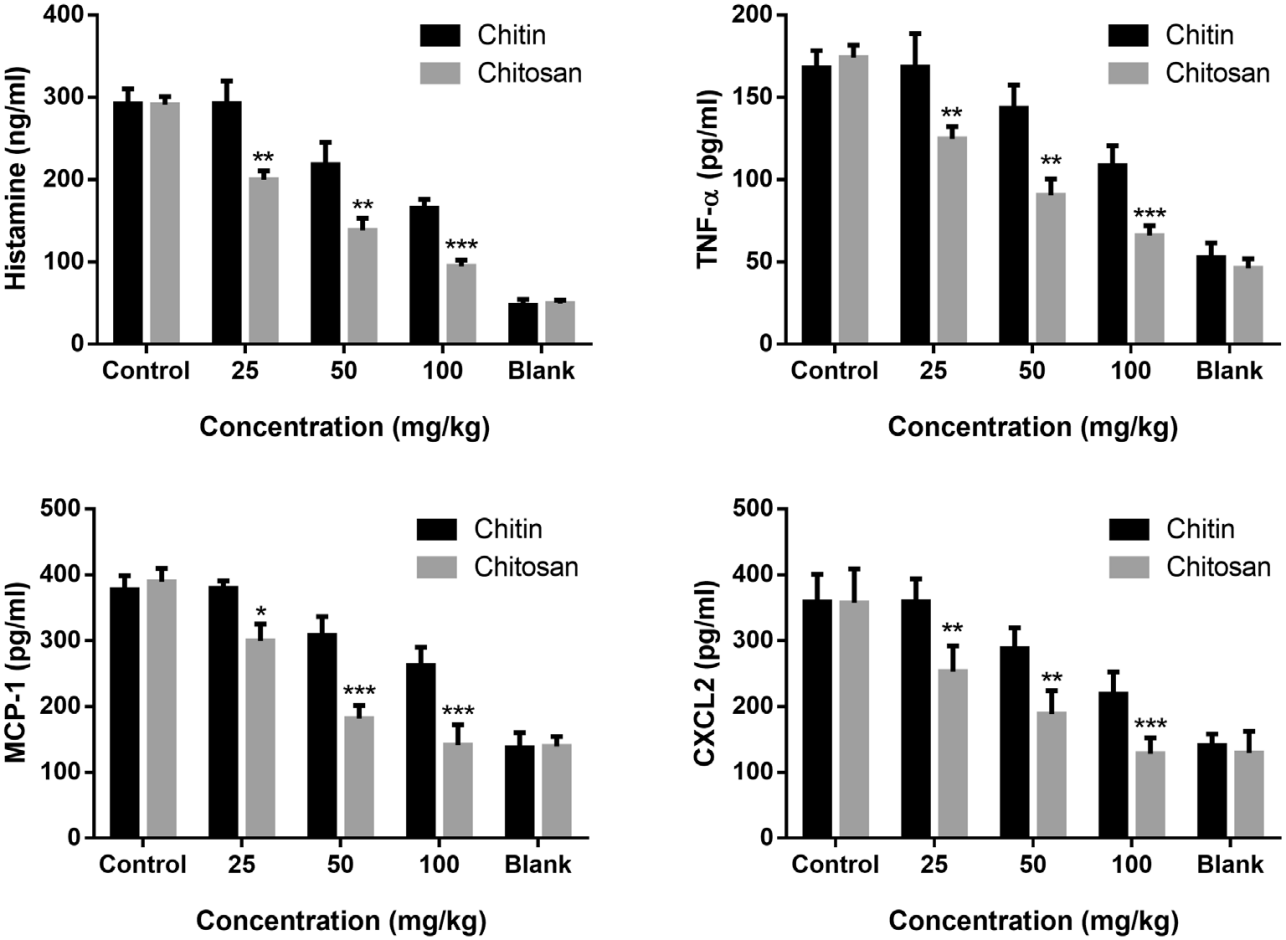
Chitin and chitosan inhibited Mrgprb2‐mediated inflammatory mediators release in mice. Chitin and chitosan inhibited SP‐induced histamine, TNF‐α, MCP‐1, and CXCL‐2 release in the serum of mice analyzed by ELISA. (*n* = 6, Data expressed as mean ± SD. Paired *t*‐test was used and differences were considered significant at *p < 0.05, **p < 0.01, and ***p < 0.005.).

Chitin and chitosan reduced the levels of histamine, TNF‐α, MCP‐1, and CXCL2 in the mice serum induced by SP, which showed that chitosan could inhibit the inflammatory reaction mediated by MRGPRX2/Mrgprb2. In addition, chitosan showed the strongest inhibition effect on inflammatory mediators release than chitin. Treated with 100 mg/kg chitin, the levels of histamine, TNF‐α, MCP‐1, and CXCL2 were 165.0 ± 11.15 ng/mL, 108.6 ± 11.97 pg/mL, 261.7 ± 28.18 pg/mL, and 218.4 ± 34.51 pg/mL respectively. At the same concentration, the levels of histamine, TNF‐α, MCP‐1, and CXCL2 were 94.81 ± 7.458 ng/mL, 66.08 ± 6.048 pg/mL, 141.5 ± 31.01 pg/mL, and 128.1 ± 24.07 pg/mL respectively after treatment with chitosan (Figure 6).

### 3.3 Chitosan Showed Stronger Inhibition on MRGPRX2 Activation Than Chitin

**FIGURE 7:**
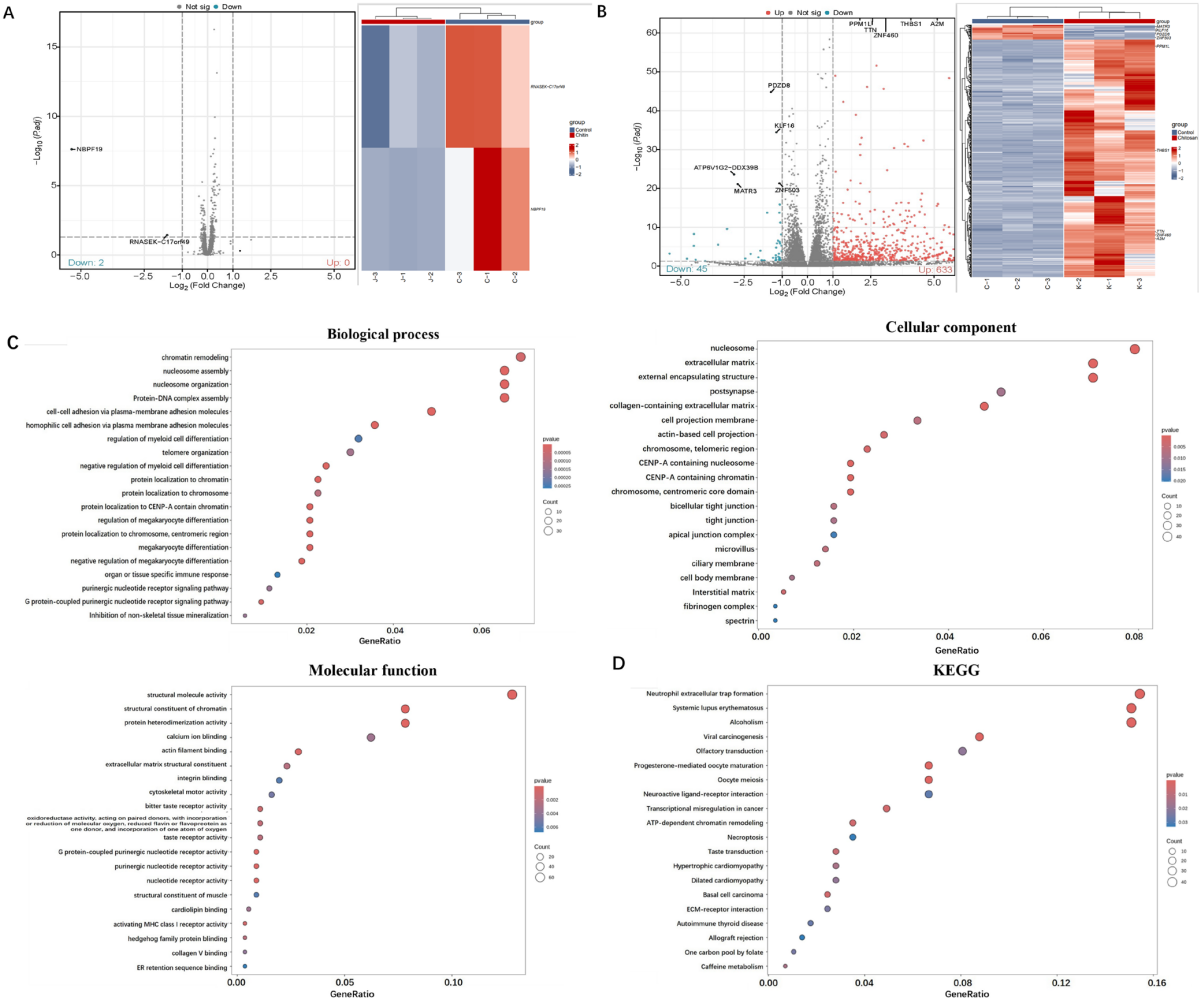
The gene expression difference in chitin and chitosan treated SP‐induced degranulation reaction model in LAD2 cells. (A) Chitin‐treated SP‐induced degranulation reaction model in LAD2 cells showed 2 down‐regulated genes compared to the control group (*n* = 3). (B) Chitosan‐treated SP‐induced degranulation reaction model in LAD2 cells showed 633 genes upregulation and 45 genes downregulation compared to the control group (*n* = 3). (C) GO enrichment analysis of chitosan‐treated SP‐induced degranulation reaction model (*n* = 3). (D) KEGG enrichment analysis of chitosan‐treated SP‐induced degranulation reaction model (*n* = 3) (the heat map and volcano icon show the top five genes with the smallest *p*‐value of differentially expressed genes between the up‐regulated and down‐regulated groups. Data were Z‐score normalized for each gene across groups).

25 μg/mL chitin and chitosan were used to treat SP‐induced MRGPRX2 activation, and the RNA‐seq was used to evaluate the inhibitory effect of chitin and chitosan on MRGPRX2 activation and to explore the mechanism of their action on MCs. Previous studies have confirmed 25 μg/mL chitosan showed significant inhibition on MRGPRX2‐mediated MC activation, while 25 μg/mL chitin showed little effect on inhibiting MRGPRX2 activation. The results showed that treatment with 25 μg/mL chitin only induced 2 genes downregulation in LAD2 cells (Figure 7A), while 25 μg/mL chitosan induced 633 genes upregulation and 45 genes downregulation (Figure 7B). So the enrichment analysis was not performed on the differential genes of chitin‐treated MCs.

However, the GO enrichment analysis of chitosan‐treated MCs showed in Figure 7C. The differential genes in biological process (BP) analysis were mainly enriched to chromatin remodeling, nucleosome assembly, nucleosome organization, protein‐DNA complex assembly, and so on. In cellular component (CC), the differential genes were mainly enriched to nucleosome, extracellular matrix, and external encapsulating structure. In addition, in molecular function (MF), the differential genes were mainly enriched to structural molecule activity, structural constituent of chromatin, and protein heterodimerization activity (Figure 7C). The KEGG analysis showed that chitosan has a major influence on the neutrophil extracellular trap formation and neuroactive ligand‐receptor interaction (Figure 7D).

**FIGURE 8:**
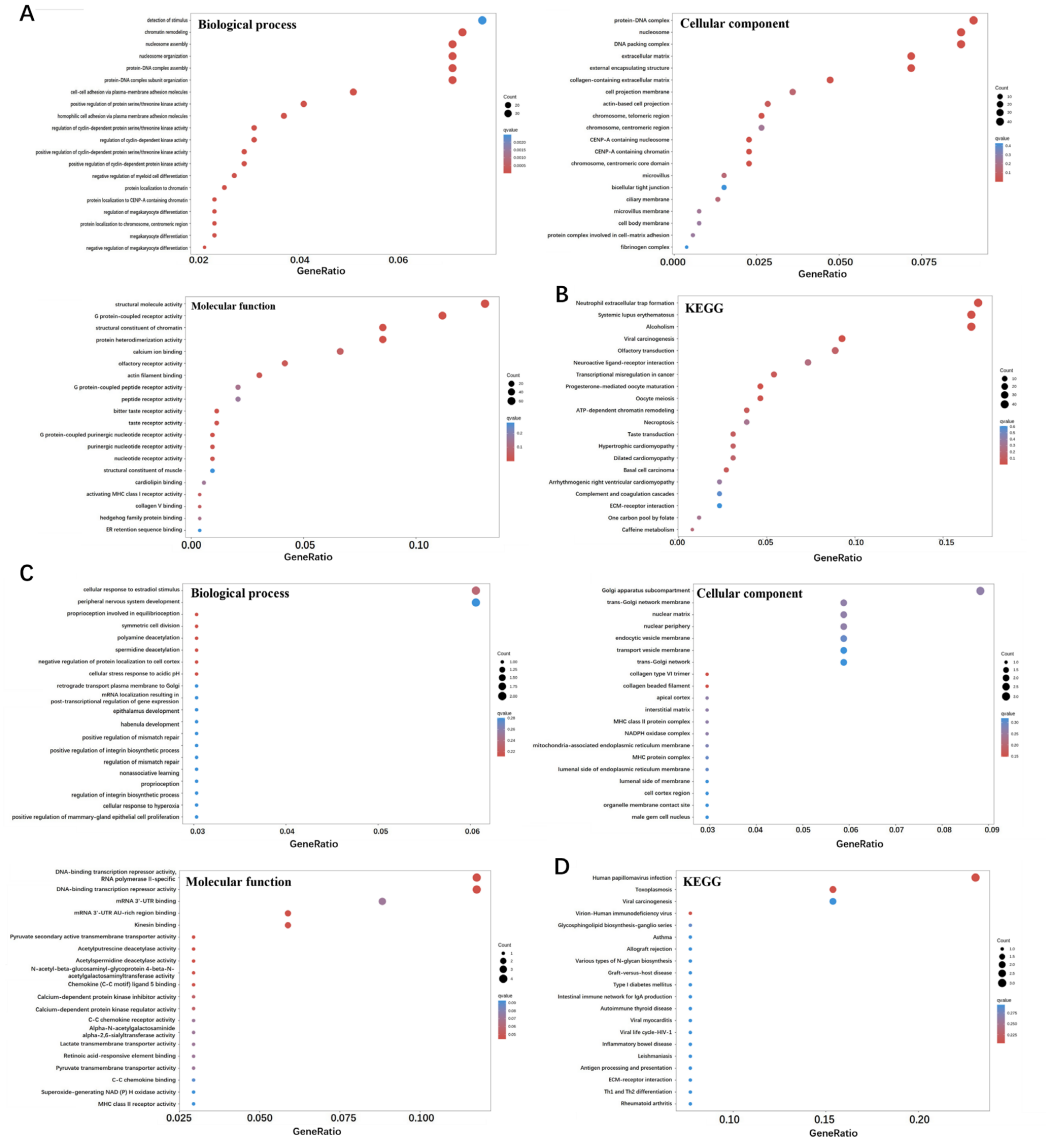
GO and KEGG enrichment analysis of upregulation genes and downregulation genes in chitosan‐treated SP‐induced degranulation reaction model in LAD2 cells. (A) GO enrichment analysis of upregulation genes (*n* = 3). (B) KEGG enrichment analysis of upregulation genes (*n* = 3). (C) GO enrichment analysis of downregulation genes (*n* = 3). (D) KEGG enrichment analysis of downregulation genes (*n* = 3).

In order to clarify the inhibitory effect of chitosan, enrichment analysis was carried out respectively for the upregulation genes and downregulation genes of chitosan‐treated LAD2 cells. GO enrichment analysis of the upregulation genes and downregulation genes is shown in Figure 8A, and the KEGG enrichment analysis of the upregulation genes is shown in Figure 8B. Because we focused on the inhibitory effect of chitosan on MRGPRX2, the downregulation genes were more important. The differential genes in biological process (BP) analysis were mainly enriched for cellular response to estradiol stimulus. In cellular component (CC), the differential genes were mainly enriched for Golgi apparatus subcompartment. In addition, in molecular function (MF), the differential genes were mainly enriched for DNA‐binding transcription repressor activity and RNA polymerase II‐specific (Figure 7C). The KEGG analysis of the downregulation genes enrichment on asthma, inflammatory bowel disease, and rheumatoid arthritis (Figure 8D). Moreover, MCs and MRGPRX2 have been proved to show an important effect on these diseases, which suggested chitosan has the potential to be used in the treatment of these MRGPRX2‐mediated related diseases. However, the specific administration methods and safety issues still require more research to clarify.

## 4 Discussion

Our data unequivocally demonstrate that both chitin and chitosan function as potent inhibitors of MRGPRX2‐ and mrgprb2‐mediated mast cell activation. This positions these naturally occurring polysaccharides as novel classes of negative regulators/antagonists for this increasingly recognized receptor, which is pivotal in IgE‐independent pseudoallergic reactions and neurogenic inflammation (Azimi et al. 2016; McNeil et al. 2015). The significantly superior inhibitory potency of chitosan over chitin observed both in vitro and in vivo warrants particular attention. This difference likely stems from their distinct physicochemical properties. Chitosan, obtained by the deacetylation of chitin, possesses abundant free amino groups that confer a strong positive charge at physiological pH. Given that many canonical MRGPRX2 agonists (e.g., substance P, icatibant, certain antimicrobial peptides) are cationic, we hypothesize that the polycationic nature of chitosan enables it to compete more effectively with these endogenous agonists for binding sites on the receptor, potentially acting as a high‐affinity competitive antagonist. Alternatively, chitosan's charge may facilitate stronger electrostatic interactions with the receptor, stabilizing an inactive conformational state or promoting receptor desensitization/internalization. Future studies employing binding assays and structural modeling are crucial to delineate the precise mode of interaction.

Chitin and chitosan, as natural derivatives, have the advantage of being nontoxic and without side effects, and both molecules and their derivatives are widely regarded as safe food additives by the U.S. Food and Drug Administration (FDA) (Wang et al. 2020; Yu et al. 2022). Depending on different intended uses, the FDA regulates the application of chitin and its derivative chitosan (Younes and Rinaudo 2015). In our work, it was found that chitin and chitosan can inhibit the activation of mast cells mediated by MRGPRX2, which may play a role in inflammatory diseases with mast cell activation. Meanwhile, due to the advantages of the physicochemical properties of chitin and chitosan, they have good potential for adjuvant therapeutic applications in diseases. Although the solubility disadvantages of chitin and chitosan affect their applications in various fields, chitosan derivatives obtained by modifying their chemical functional groups can overcome these disadvantages. Chitosan and its derivatives show good solubility and pH‐sensitive targeting, as well as good bioactivity, biocompatibility, biodegradability, and mucosal adhesion properties. In addition, chitin and chitosan possess original bactericidal, antimicrobial, anticancer, and other effects, as well as the ability to induce erythrocyte aggregation, promote platelet activation, and activate the complement system (Christou et al. 2019; Iftime et al. 2019; Kritchenkov et al. 2019; Pavoni et al. 2019). Chitosan plays an important role in food preservation and packaging because of its good film‐forming properties and antimicrobial and antioxidant effects. Moreover, chitosan's properties are useful for avoiding the addition of chemical preservatives to food and producing edible antimicrobial films (Friedman and Juneja 2010; Lv et al. 2023).

In the present study, chitosan was able to inhibit MrgprX2‐mediated mast cell activation, possibly due to protonated amino groups of chitosan (cationic charge). In general, positively charged substances are able to activate MRGPRX2, and chitosan may competitively inhibit the binding of other positively charged activators to MRGPRX2, thereby exerting an inhibitory effect (Kühn et al. 2021). Compared to chitin properties, chitosan and its derivatives have better solubility, hydrophilicity, and pH sensitivity; they are soluble in acidic aqueous solutions and can be used to prepare hydrogels and other composites with better bioavailability (Younes and Rinaudo 2015). In order to make a more intuitive comparison, the working concentration of chitosan was selected for RNA‐seq, and the same concentration of chitin was selected for sequencing. Because chitin is less effective in inhibiting mast cell degranulation, it is possible that chitin has not yet shown its inhibitory effect at the concentration at which chitosan begins to exert its effect, which is the reason for only two differential genes.

Fundamental physicochemical differences between these two polymers, particularly their solubility and charge properties (governed by pKa), directly impact their bioavailability and molecular interactions with the MRGPRX2 receptor. Firstly, solubility is a critical determinant of bioavailability. Chitin, characterized by its highly crystalline structure and extensive inter‐ and intra‐molecular hydrogen bonding via acetyl groups, exhibits very poor solubility in aqueous media at physiological pH (Younes and Rinaudo 2015). This inherent insolubility severely limits the amount of chitin molecules available in solution to interact with mast cells or the MRGPRX2 receptor in our assays. In contrast, chitosan, resulting from the partial deacetylation of chitin, possesses abundant free amino groups. This structural modification drastically reduces crystallinity and enhances hydrophilicity, rendering chitosan soluble in acidic aqueous environments commonly used in biological buffers (including those in our in vitro studies) and potentially within inflammatory microenvironments in vivo. Secondly, and perhaps more crucially for receptor interaction, is the difference in charge stemming from their distinct pKa values. The free amino groups on chitosan have a pKa typically ranging from 6.3 to 6.8 (Younes and Rinaudo 2015). At physiological pH (pH ~7.4) and within the slightly acidic milieu often associated with inflammation or certain experimental conditions, a significant proportion of these amino groups become protonated (−NH_3_^+^), conferring chitosan a polycationic nature. In stark contrast, chitin, retaining its N‐acetyl groups, remains largely uncharged and neutral at physiological pH. Its lack of cationic charge significantly diminishes its ability to compete with cationic agonists or engage electrostatically with critical regions of the MRGPRX2 receptor, explaining its weaker inhibitory activity despite structural similarity to chitosan.

Our RNA‐seq analysis, while primarily exploratory, provides valuable initial insights into the potential molecular underpinnings of chitosan's inhibitory action. The significant enrichment of terms related to ‘structural molecule activity,’ ‘chromatin architectural component,’ and ‘protein heterodimerization activity’ among the differentially expressed genes is particularly intriguing in the context of MRGPRX2 signaling. Structural molecule activity alterations could reflect interference with cytoskeletal reorganization, a process essential for degranulation, or impact the scaffolding proteins within the MRGPRX2 signalosome. Changes in chromatin architectural components suggest that chitosan might exert longer‐term effects on mast cell function by modulating chromatin accessibility and transcriptional programs governing inflammatory responses or even receptor expression itself, which could be highly relevant for chronic conditions. Most pertinently, the modulation of protein heterodimerization activity strongly hints at interference with the assembly of critical signaling complexes downstream of MRGPRX2 activation. This receptor relies on coupling to heterotrimeric G proteins (notably Gαq) and recruitment of β‐arrestins for initiating distinct signaling cascades leading to degranulation and cytokine production (Pundir et al. 2019). Chitosan might disrupt the formation or stability of these receptor‐G protein or receptor‐β‐arrestin heterodimers, thereby effectively blunting signal transduction. Validating these potential mechanisms, focusing on specific candidate genes and protein interactions identified by the RNA‐seq data (e.g., via co‐immunoprecipitation or proximity ligation assays), represents a key future direction.

Chitin and chitosan can be administered orally, topically, or by injection, either directly or through transmitters. Oral administration is the most convenient, safe, and efficacious mode of administration (Shabani et al. 2023). Simultaneously, chitosan can improve the efficacy of drugs by promoting interactions between drugs and target cells (Sangnim et al. 2023; Singh et al. 2016). Moreover, they can improve intestinal barrier function and increase beneficial intestinal microbiota, thereby alleviating systemic diseases, especially cardiovascular diseases such as atherosclerosis and inhibiting oxidative stress‐induced aging (Anandan et al. 2013; Morganti et al. 2012; Y. Yu et al. 2015; Zhu et al. 2020). Additionally, chitin and chitosan have the potential to improve respiratory, renal, endocrine, infectious, and inflammatory disease treatments.

Nonetheless, chitin presents the two sides of the coin in terms of the inflammatory response. On the one hand, chitin nanofibers inhibit skin inflammation in mice with Alzheimer's disease (Izumi et al. 2016). On the other hand, it has also been suggested that chitin activates type 2 innate lymphoid cells and γδT cells, thereby triggering an inflammatory response (Van Dyken et al. 2014). In addition, chitin was reported to activate dendritic cells in an ovalbumin‐induced mouse model and promote the release of IL‐33 (Arae et al. 2018). This is in contrast to the inhibition of mast cell activation by chitin in our study, and its complex role in the inflammatory response also presents a two‐sided effect. This suggests that chitin exerts multiple effects in different disease models, possibly by acting on different cell types. Interestingly, Wagener et al. demonstrated that chitin could activate anti‐inflammatory cytokines such as IL‐10 via NOD2 and Toll‐like receptor (TLR)‐9 activation. This suggests that chitin can regulate the inflammatory process by triggering inflammatory and anti‐inflammatory responses in a negative feedback‐regulated manner. Its anti‐inflammatory properties may be related to its concentration (Wagener et al. 2014). It has also been suggested that chitin nanofibrils and chitin nanorod structures can downregulate proinflammatory cytokines, while upregulating the antimicrobial peptide β‐defensin 2 in human keratinocytes (Danti et al. 2019). Chitosan and its derivatives have also been reported as anti‐inflammatory agents capable of stimulating the expression of the tight junction proteins claudin‐1, occludin, and ZO‐1, and restoring intestinal barrier function in DSS‐induced colitis (J. Wang et al. 2019). Oral administration of oligochaetes attenuates acute and chronic colitis in mice by inhibiting the NF‐κB pathway and downregulating its downstream target COX‐2 and upstream target TLR‐4 (Tao et al. 2022; Yousef et al. 2012). Furthermore, it has been found that chitosan oligosaccharides protect against shrimp protomyosin‐induced food allergy by downregulating IL‐4, IL‐5, and IL‐13 and upregulating interferon (IFN)‐γ (Jiang et al. 2019). Activation of MCs by MRGPRX2 may mediate neurogenic inflammation, pain, and pruritus. MRGPRX2 is an important target for mediating the pathogenesis of a variety of diseases, including atopic dermatitis (Jia et al. 2024), chronic spontaneous urticaria (Fujisawa et al. 2014), pseudoallergic reactions (McNeil et al. 2015), hypersensitivity‐type inflammation (Green et al. 2019), and rosacea (Muto et al. 2014).

Our study showed that chitin and chitosan effectively inhibited the activation of MRGPRX2 in MCs both in vitro and in *vivo*. The potent MRGPRX2 inhibitory activity of chitosan, coupled with its favorable safety profile and established use in dietary supplements (e.g., for lipid management), positions it exceptionally well for development as a functional food ingredient or nutraceutical. This approach holds significant promise for the prevention or adjunctive management of MRGPRX2‐mediated chronic diseases. For instance, oral chitosan supplementation could modulate mast cell activity in the gut or systemically, potentially benefiting conditions like chronic urticaria or systemic mast cell activation syndromes. Topical formulations containing chitosan could be explored for localized conditions like atopic dermatitis or rosacea, leveraging its anti‐inflammatory and potentially barrier‐enhancing properties alongside MRGPRX2 inhibition. Realizing this potential will require further investigation into optimal chitosan specifications (DD, MW), formulation strategies for bioavailability/stability, and rigorous evaluation in relevant chronic disease models.

## 5 Conclusion

In conclusion, our study suggested that chitin and chitosan could inhibit MRGPRX2‐mediated MC degranulation reaction and cytokines release in vitro. Chitin and chitosan reduced SP‐induced local passive anaphylaxis by inhibition of Mrgprb2‐mediated MC activation in vivo. RNA‐seq analysis indicated that it may affect the activity of structural molecules, chromatin structural components, and protein heterodimerization activity. Chitosan showed a stronger inhibition effect on MRGPRX2 activation than chitin. Chitosan has the potential to be developed into functional foods to assist in the treatment of MRGPRX2‐mediated chronic diseases. Of course, this depends on further studies of oral pharmacokinetics, bioavailability, or intestinal tolerance.

## Author Contributions

**Tao Jia:** conceptualization (equal), formal analysis (equal), investigation (equal), methodology (equal), resources (equal), software (equal), writing – original draft (equal), writing – review and editing (equal). **Ruiqi Li:** conceptualization (equal), data curation (equal), investigation (equal), writing – original draft (equal). **Chenkun Liu:** conceptualization (equal), formal analysis (equal), investigation (equal), methodology (equal), writing – review and editing (equal). **Yifan Xia:** conceptualization (equal), investigation (equal), methodology (equal), supervision (equal). **Mengyao Yi:** conceptualization (equal), investigation (equal), methodology (equal), supervision (equal), writing – review and editing (equal). **Xiangjin Song:** investigation (equal), methodology (equal), supervision (equal). **Tong Zhou:** investigation (equal), methodology (equal), supervision (equal). **Delu Che:** conceptualization (equal), investigation (equal), methodology (equal), resources (equal), software (equal), writing – original draft (equal), writing – review and editing (equal). **Ning Kong:** conceptualization (equal), formal analysis (equal), investigation (equal), methodology (equal), resources (equal), software (equal), writing – review and editing (equal).

## Ethics Statement

This study was carried out in strict accordance with the recommendations in the Guide for the Care and Use of Laboratory Animals from the National Institutes of Health.

## Consent

The authors have nothing to report.

## Conflicts of Interest

The authors declare no conflicts of interest.

## Data Availability

The original RNA‐seq data has been uploaded to the NCBI SRA database (https://www.ncbi.nlm.nih.gov/bioproject/?term=PRJNA1179930, BioProject ID: PRJNA1179930).
